# Acute Activation, Desensitization and Smoldering Activation of Human Acetylcholine Receptors

**DOI:** 10.1371/journal.pone.0079653

**Published:** 2013-11-14

**Authors:** Barbara G. Campling, Alexander Kuryatov, Jon Lindstrom

**Affiliations:** 1 Department of Neuroscience, University of Pennsylvania, Philadelphia, Pennsylvania, United States of America; 2 Department of Medical Oncology, Thomas Jefferson University, Philadelphia, Pennsylvania, United States of America; Duke University Medical Center, United States of America

## Abstract

The behavioral effects of nicotine and other nicotinic agonists are mediated by AChRs in the brain. The relative contribution of acute activation versus chronic desensitization of AChRs is unknown. Sustained “smoldering activation” occurs over a range of agonist concentrations at which activated and desensitized AChRs are present in equilibrium. We used a fluorescent dye sensitive to changes in membrane potential to examine the effects of acute activation and chronic desensitization by nicotinic AChR agonists on cell lines expressing human α4β2, α3β4 and α7 AChRs. We examined the effects of acute and prolonged application of nicotine and the partial agonists varenicline, cytisine and sazetidine-A on these AChRs. The range of concentrations over which nicotine causes smoldering activation of α4β2 AChRs was centered at 0.13 µM, a level found in smokers. However, nicotine produced smoldering activation of α3β4 and α7 AChRs at concentrations well above levels found in smokers. The α4β2 expressing cell line contains a mixture of two stoichiometries, namely (α4β2)_2_β2 and (α4β2)_2_α4. The (α4β2)_2_β2 stoichiometry is more sensitive to activation by nicotine. Sazetidine-A activates and desensitizes only this stoichiometry. Varenicline, cytisine and sazetidine-A were partial agonists on this mixture of α4β2 AChRs, but full agonists on α3β4 and α7 AChRs. It has been reported that cytisine and varenicline are most efficacious on the (α4β2)_2_α4 stoichiometry. In this study, we distinguish the dual effects of activation and desensitization of AChRs by these nicotinic agonists and define the range of concentrations over which smoldering activation can be sustained.

## Introduction

The component of tobacco that drives its compulsive use is nicotine, an alkaloid that acts on nicotinic acetylcholine receptors (AChRs) in the brain. AChRs are a heterogeneous family of ligand-gated cation channels which consist of five homologous subunits arranged around a central pore [1], [2]. They are heteropentamers formed of combinations of α and β subunits, or homopentamers formed entirely of α7 subunits [3]. Heteromeric AChRs usually have two ACh binding sites that are formed between adjacent α and β subunits. The remaining subunit is in the “accessory” position. While the accessory subunit does not usually form part of a binding site for ACh, it has major effects on responses to nicotinic agonists, antagonists and allosteric modulators. There is a third ACh binding site in the (α4β2)_2_α4 stoichiometry, formed at the interface between the α4 accessory subunit and another α4 subunit [4], [5].

The predominant AChR subtypes in human brain are heteromeric AChRs comprised of combinations of α4 and β2 subunits, alone or in combination with other subunits, such as β3, α5 or α6, or homomeric AChRs comprised of α7 subunits. Self-administration of nicotine is inhibited by knockout of α4, β2, or α6 subunits [6], but is increased by knockout of α5 subunits [7]. AChRs containing the α3 subunit are the predominant postsynaptic AChRs in the autonomic and enteric nervous systems [8]. In the brain, α3 and β4 subunits are prominent only in the medial habenula [9].

Although nicotine is a drug of abuse, it also has many positive effects that could be exploited therapeutically. In addition to their use for treating nicotine addiction, nicotinic agonists are being developed for treatment of disorders ranging from Alzheimer’s disease to schizophrenia [10]. Varenicline and cytisine have been promoted for treating nicotine addiction because they are high affinity partial agonists that displace nicotine and decrease its rewarding effects by attenuating dopamine release in the mesolimbic system [11]. However, many of the clinical effects of nicotine and partial agonists are mediated by desensitization of AChRs. Sazetidine-A is a very potent and high affinity acute agonist, and a very efficient desensitizer. It produces many of the *in vivo* effects of nicotine and partial agonists such as inhibition of nicotine self-administration, increased attention, pain relief, reduction in anxiety and depression, and weight reduction, suggesting that these effects may result more from desensitization than from activation [12]–[17].

All of these cholinergic ligands upregulate (α4β2)_2_β2 AChRs in tissue culture, and all but sazetidine-A also do so *in vivo* [18]. Sazetidine-A may have sufficient membrane permeability *in vivo* to desensitize AChRs on neuron surfaces, but insufficient to achieve intracellular concentrations sufficient to promote assembly of (α4β2)_2_β2 AChRs [18], [19].

Transient application of nicotine or other agonists activates AChRs, opening the cation channel resulting in depolarization and other effects mediated by entry of cations, followed by acute desensitization and then rapid recovery. Chronic exposure to agonists causes prolonged desensitization. In typical physiological assays, the effects of these drugs are observed over seconds or minutes. However, *in vivo* they are present over many hours. “Smoldering activation” occurs at agonist concentrations at which some AChRs are desensitized while others are activated. This can occur within minutes after recovery from acute activation or after prolonged continuous exposure to agonists. The relative contributions of acute activation and chronic desensitization of AChRs in causing as well as treating nicotine addiction, or mediating the beneficial effects of nicotine are being actively investigated [18], [20].

We have established a number of transfected cell lines that permanently express human AChR subtypes [21]–[25]. The α4β2 expressing cell line contains a mixture of two stoichiometries differing by the presence of α4 or β2 in the accessory position [22]. A similar mixture of stoichiometries is found in brain [26], [27]. The (α4β2)_2_β2 stoichiometry is more sensitive to activation and upregulation by nicotine, desensitizes more slowly and is less permeable to calcium [22], [28]. It is sensitive to activation by sazetidine-A, but not varenicline or cytisine, whereas the (α4β2)_2_α4 stoichiometry is sensitive to activation by varenicline and cytisine but not sazetidine-A [29], [30].

It is difficult to measure chronic desensitization of human AChRs using classical electrophysiological techniques such as human AChRs expressed in *Xenopus* oocytes or patch clamp studies on individual cells. Furthermore, until recently, it has not been possible to express sufficient amounts of α7 AChRs in human cell lines for functional assays. To obtain sufficient expression of α7 AChRs, we used chemical chaperones to promote assembly of human α7 in a cell line which co-expresses α7 and the AChR chaperone protein RIC-3 [25].

In this study, we examined the effects of acute and prolonged application of nicotine and three drugs which are known to inhibit nicotine self-administration (varenicline, cytisine and sazetidine-A), on human α4β2, α3β4 and α7 AChRs. These studies confirm and extend basic expectations of the properties of these agonists. The agonists fully desensitized these AChR subtypes with the exception of sazetidine-A on (α4β2)_2_α4. We have defined the range of concentrations of each of these agonists which can sustain smoldering activation of these AChRs. For α4β2, but not α3β4 or α7 AChRs, smoldering activation occurs at concentrations of nicotine that are sustained in smokers.

## Methods

### cDNAs and Chemicals

Human α3, α4, α7, β2 and β4 cDNAs were cloned in this laboratory as described previously [21], [31], [32]. Sazetidine-A was from Tocris Bioscience (Ellisville, MO). All other chemicals were from Sigma-Aldrich (St. Louis, MO).

### Cell Lines and Transfection

The parental cell line used for transfections was tsA201, derived from the human embryonic kidney cell line (HEK) 293 [33]. The cell line permanently expressing α4β2 AChRs [22], [23] has a mixture of two α4β2 stoichiometries, namely (α4β2)_2_α4, which has lower sensitivity, and (α4β2)_2_β2, which has higher sensitivity to activation by ACh [22]. The cell line permanently expressing α3β4 AChRs has been described [21], [34]. A stable cell line expressing functional α7 AChRs was prepared by initially transfecting the tsA201 cell line with cDNA for α7 and subsequently transfecting with cDNA for the AChR-selective chaperone, human RIC-3. The expression of functional α7 AChRs in this cell line was further increased by growth in the chemical chaperones valproic acid (VPA, 1 mM) and 4-phenylbutyric acid (PBA, 3 mM) for at least 2 weeks before functional assays [25].

All transfected cell lines were grown in Dulbecco’s modified Eagle’s medium (InVitrogen, Carlsbad, CA) with 10% fetal bovine serum (Hyclone, Logan, UT) supplemented with 2 mM glutamine. The expression of α4, α3 and α7 was maintained with Zeocin (0.5 mg/ml) and the expression of β2, β4 and RIC-3 was maintained with G418 (0.6 mg/ml). The cell lines were grown at 37°C with 5% CO_2_ in a humidified atmosphere.

### Acute Activation

Responses to nicotinic agonists were determined using a FLEXStation microplate fluorometer (Molecular Devices, Sunnyvale, CA) as described [23]. For cell lines expressing α4β2 and α3β4 AChRs, the cells were plated at 10^5^ cells/ml (100 µl/well) in black-walled clear-bottom 96 well plates (Costar, Fisher Scientific, Pittsbugh, PA), and incubated for 48 hours prior to assaying responses to various nicotinic agonists. The α7/RIC-3 expressing cells were plated at 5×10^5^/ml in black-walled clear-bottom 96 well BioCoat plates (BD Biosciences, Franklin Lakes, NJ) in the continued presence of VPA and PBA, with the addition of 5% human AB serum (Pel-Freez Biologicals, Rogers, AR) to further increase expression of α7 [35]. The cells were then grown for 24 hours prior to the assay.

To measure responses to various nicotinic agonists, 100 µl of a fluorescent dye which is sensitive to changes in membrane potential (Molecular Devices) was added to the wells. The dye was prepared according to the manufacturer’s instructions, with the addition of atropine (0.5 µM) to block muscarinic responses. The plates were then incubated at 37°C for one hour prior to the assay. Serial dilutions of agonists were then prepared in Hanks Balanced Salt Solution (HBSS) in V-shaped 96-well plates (Fisher Scientific Co, Pittsburgh, PA). Fluorescent responses were measured in the FLEX Station at 25°C, with an excitation wavelength of 530 nm and an emission wavelength of 565 nm. Various agonists (50 µl) were added after the first 20 seconds and responses were followed for 60–120 seconds.

### Chronic Desensitization

To measure desensitization, plates were prepared as for agonist assays. Sixteen hours prior to the assay, serial dilutions of agonists, prepared in regular growth medium, were added to the plates. One hour before the assay, the membrane potential-sensitive fluorescent dye with 0.5 µM atropine was added to the wells. For α4β2 AChRs, responses were measured to 3 µM ACh (to detect responses of (α4β2)_2_β2 AChRs) and 100 µM ACh (to detect responses of both stoichiometries). For α3β4 and α7 AChRs, responses to saturating concentrations of ACh (1 mM for α3β4 and 10 µM for α7) were measured. Each data point represents the average of peak values for individual dose response curves from 4–8 wells.

### Expression of (α4β2)_2_β2 AChRs

To determine which stoichiometry of α4β2 AChRs was affected by sazetidine-A, we performed short term transfection of β2 subunits into the α4β2 expressing cell line. To increase the expression of the higher sensitivity (α4β2)_2_β2 stoichiometry, the cell line expressing α4 and β2 was cotransfected with human β2 (pRc-CMV/Geneticin) using the FuGene6 transfection agent (Roche Diagnostics, Indianapolis, IN). On the following day, 0.5 µM nicotine was added to further increase the expression of the (α4β2)_2_β2 stoichiometry. After incubation for 24 hr with nicotine, the cells were washed twice with growth medium, and then serial dilutions of sazetidine-A were added for 6 hours prior to the assay. Desensitization by sazetidine-A was measured using the FlexStation as described above, with 3 µM and 100 µM ACh.

### Smoldering Activation following Acute Desensitization

Responses of α4β2, α3β4 and α7 AChRs to the acute application of ACh (100 µM), nicotine (16 µM), varenicline (4 µM), cytisine (16 µM) and sazetidine-A (62.5 nM) were measured using the FlexStation as described. These drug concentrations were selected because they gave maximum sustained responses to these agonists. Results were expressed as a percentage of maximum response to ACh. Responses were monitored for 10 minutes, and then specific antagonists were added and responses recorded for another two minutes. The antagonists were dihydroβerythroidine (DHβE) (1 µM) for α4β2, mecamylamine (MCA (10 µM) for α3β4 and methyllycaconitine (MLA) (10 µM) for α7. These concentrations of antagonists were selected because they were sufficient to inhibit responses to the tested agonists without causing activation themselves.

### Statistics

Data were graphed using GraphPad Prism software. Non-linear models (sigmoidal dose-response with variable slope or two site competition) were used to fit the concentration response curves. The EC50 and IC50 were calculated from the curves. Kaleidagraph software was used to determine Hill slopes and standard errors of the EC50 and IC50.

## Results

### Acute Responses to Nicotinic Agonists

**Figure 1:**
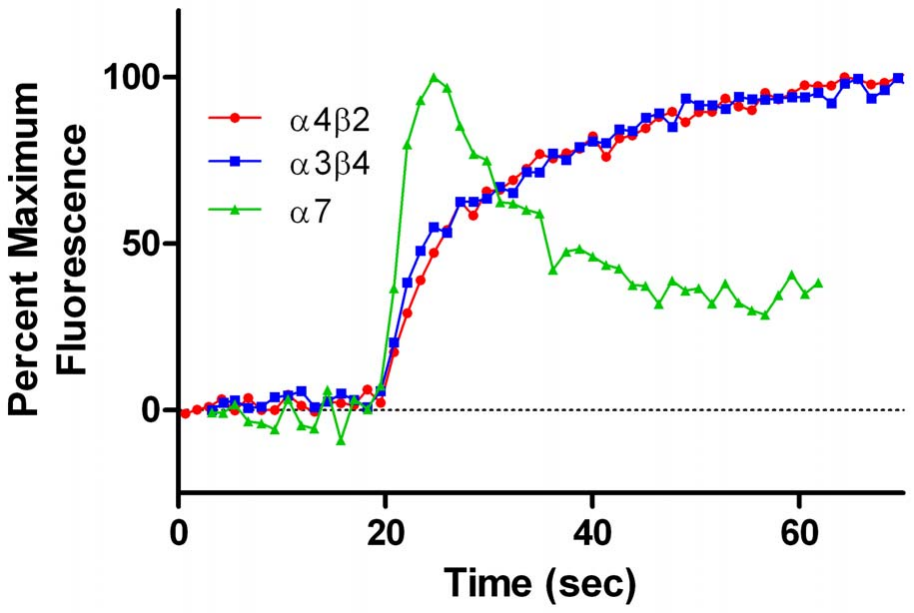
Acute responses of AChRs to application of saturating concentrations of ACh. Fluorescent responses were measured using the FLEXStation with a membrane potential-sensitive indicator. The kinetics of responses of α4β2 (to 300 µM ACh) and α3β4 AChRs (to 1.0 mM ACh) were very similar, with a maximum response reached within 45 seconds of agonist application. The response of α7 AChRs (to 10 µM ACh) was more rapid, with a peak response within 5 seconds, followed by rapid desensitization. Each data point represents the average of 4 individual response curves. The absolute values of responses of saturating concentrations of ACh (expressed as relative fluorescence units) were similar for α4β2 (167,000+/−18,000) and α3β4 (161,000+/−14,000), but significantly lower for α7 AChRs (54,00+/−3000), probably as a result of rapid desensitization.

Acute responses of α4β2, α3β4 and α7 AChRs were tested after application of a range of concentrations of ACh, nicotine, varenicline, cytisine and sazetidine-A, using the FlexStation with an indicator which is sensitive to changes in membrane potential. Responses of these AChRs to saturating concentrations of ACh are shown in Figure 1. The kinetics of responses of the other agonists were very similar to those of ACh (data not shown). The effects of saturating concentrations of ACh on α4β2 and α3β4 AChRs had the same appearance, with a maximum response reached within 40 seconds of agonist application. The responses of α7 AChRs were quite different with a maximum response within 5 seconds, followed by acute desensitization. However, the rate of acute desensitization was less than detected with electrophysiological techniques [36]–[38]. The amplitudes of responses of saturating concentrations of ACh (expressed as relative fluorescence units) were similar for α4β2 (167,000+/−18,000) and α3β4 (161,000+/−14,000), but significantly lower for α7 AChRs (54,000+/−3000), probably as a result of rapid desensitization [36].

**Figure 2:**
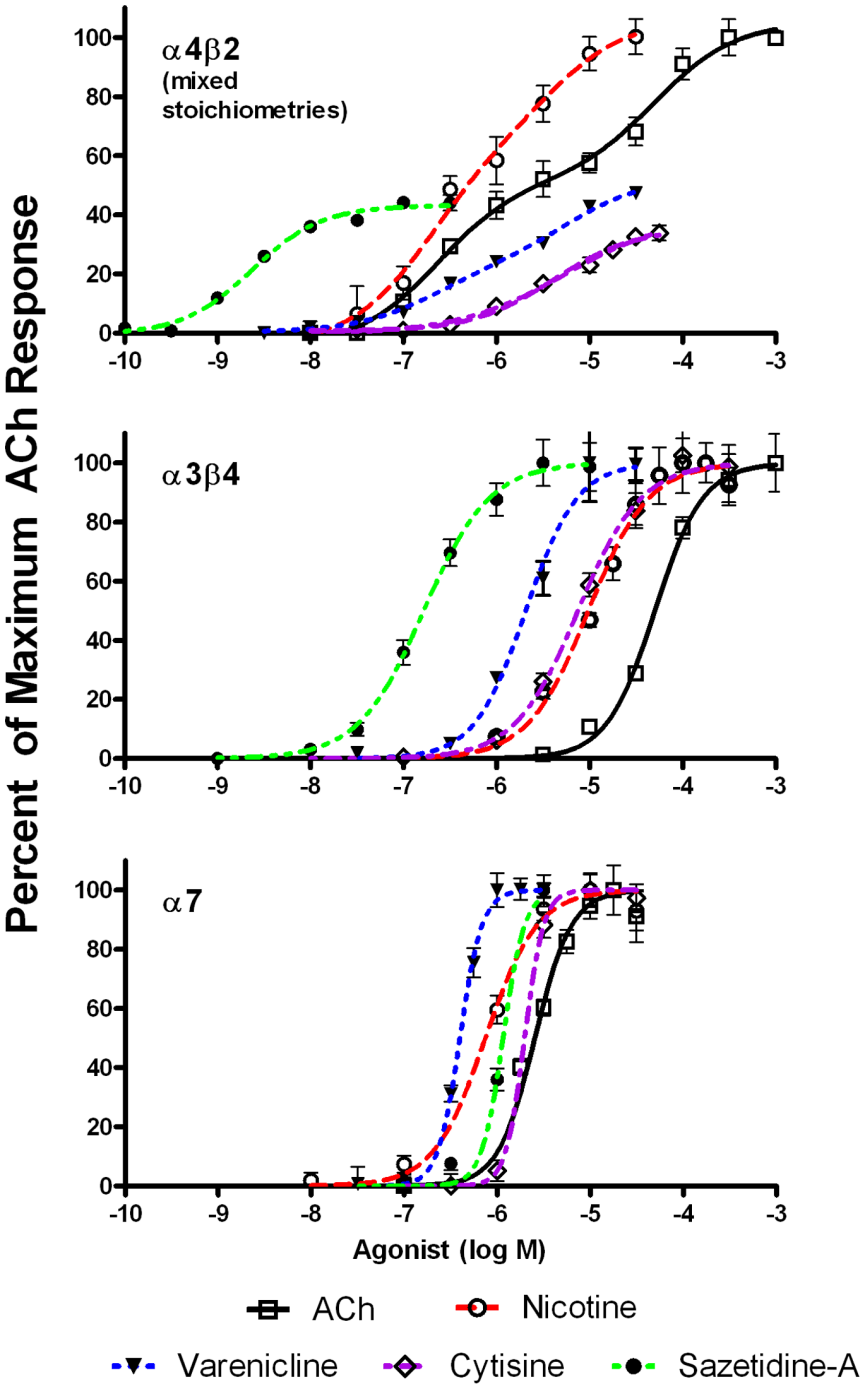
Responses of human α4β2, α3β4 and α7-expressing cell lines to various concentrations of nicotinic agonists. Responses were measured using the FLEXStation with an indicator sensitive to changes in membrane potential. Results are expressed as a percent of maximal fluorescence. Each data point is an average of the peak fluorescence of 4–8 individual dose-response curves. Nicotine and ACh are full agonists on α4β2, whereas varenicline, cytisine and sazetidine-A are partial agonists. All of the tested compounds are full agonists on α3β4 and α7 AChRs.

The concentration response curves for various agonists on α4β2, α3β4 and α7 AChRs are shown in Figure 2. The EC50’s are summarized in Table 1. Nicotine had an efficacy comparable to that of ACh on α4β2* AChRs, whereas varenicline, cytisine and sazetidine-A were partial agonists, with efficacies of 48% for varenicline, 34% for cytisine and 44% for sazetidine-A. All of the tested compounds were full agonists on α3β4 and α7 AChRs. The EC50 values for α7 were lower than often reported by electrophysiological techniques. The EC50 values for α7 vary widely according to the assay method [36], [39]–[41]. Millisecond agonist kinetics are probably irrelevant for drugs present in the body for hours. It is likely that sustained smoldering activation and sustained antagonism due to desensitization are the most relevant factors. The fluorescence indicator is a more sensitive measure of α7 activation, although the response kinetics are slower [38].

**Table 1:** Agonist Efficacy and Sensitivity for Activation and Desensitization.

	ACh		Nicotine		Varenicline		Cytisine		Sazetidine-A	
Activation EC_50_ (µM)		Hill		Hill		Hill		Hill		Hill
α4β2	0.23±0.05	1.5	0.18±0.03	1.8	0.20±0.1	1.1	5.5±0.8	0.9	0.0023±0.0003	1.2
	51±35	1.3	4.2±0.5	2.5	6.1±0.2	1.8				
α3β4	50±3.6	1.8	9.7±1.3	1.1	2.1±0.3	1.4	7.5±1.1	1.3	0.17±0.03	1.3
α7	2.5±0.3	2.4	0.75±0.1	1.7	0.4±0.02	3.5	2.0±0.06	4.3	1.2±0.08	1.6
**Efficacy (%) (relative to ACh)**										
α4β2	–		100		48		34		44	
α3β4	–		100		100		100		100	
α7	–		100		100		100		100	
**Desensitization IC_50_ (µM)**										
α4β2: 3 µM ACh	–		0.04±0.004	−1.0	0.02±0.003	−0.6	0.001±0.0005	−1.1	0.00048±0.00002	−0.9
							0.25±0.1	−1.1		
α4β2: 100 µM ACh	–		5.0±1.0	−1.2	0.9±0.08	−1.0	0.003±0.0003 20±7.0	−1.2	0.00026±0.0002	−0.6
								−0.8		
α3β4: 1.0 mM ACh	–		9.1±1.3	−0.9	2.5±0.4	−1.1	1.3±0.5	−0.6	0.33±0.1	−0.5
α7: 10 µM ACh	–		3.4±0.18	−3.2	0.4±0.05	−2.6	4.2±0.74	−1.4	3.5±0.4	−2.9
**Intercept of Activation and Desensitization Curves** **(µM)**										
α4β2: 3 µM ACh	–		0.13		0.16		0.45		0.0015	
α4β2: 100 µM ACh	–		1.82		0.93		0.74		0.002	
α3β4: 1.0 mM ACh	–		8.7		2.1		4.1		2.2	
α7: 10 µM ACh	–		1.7		0.4		2.1		1.5	

The EC50’s for activation of α4β2, α3β4 and α7 AChRs are expressed in µM. In cases where the dose response curves fit with a two-site competition model, the EC50 for the higher sensitivity component is listed first. For desensitization, cell lines expressing various human AChRs were incubated overnight in the presence of a range of concentrations of agonists, and then tested for activation by ACh. For α4β2 AChRs, two concentrations of ACh were tested, namely 3 µM (to test the more sensitive stoichiometry (α4β2)_2_β2), and 100 µM ACh (to assay function of both stoichiometries). For the other AChRs, saturating concentrations of ACh were used (1.0 mM for α3β4 and 10 µM for α7).

The cell line transfected with α4 and β2 subunits expresses a mixture of (α4β2)_2_β2 and (α4β2)_2_α4 stoichiometries, which have different sensitivities to nicotine and other agonists. The concentration response curves for acetylcholine, nicotine and varenicline fit with a two-site competition model, likely indicating that these agonists have different effects on the two stoichiometries. On the other hand, the dose response curves for cytisine and sazetidine-A were monophasic, likely because these agonists act on only one stoichiometry.

The EC50 of nicotine for the more sensitive (α4β2)_2_β2 stoichiometry was 0.18 µM, which is within the range of nicotine levels detected in smokers (see Discussion). For varenicline, the EC50 for the more sensitive stoichiometry was 0.20 µM, which is close to the peak blood levels of 0.1 µM detected in humans after a 1 mg dose of this drug [42]. On the other hand, the EC50 for nicotine on α3β4 was 9.7 µM and for α7 AChRs was 0.75 µM, levels which cannot be reached in the systemic circulation. The EC50 for varenicline on α7 AChRs was 0.4 µM, which is close to levels reached in humans after a dose of 1 mg [42]. The EC50 of cytisine for α4β2 AChRs was 5.5 µM. It is uncertain whether this is a clinically achievable level. Sazetidine-A was the most potent of all the agonists on α4β2 AChRs (EC50 = 0.0023 µM). In mice treated with 2 mg/kg sazetidine-A, serum levels of 1.6 µM and brain levels of 0.15 µM are reached [19].

### Desensitization

**Figure 3:**
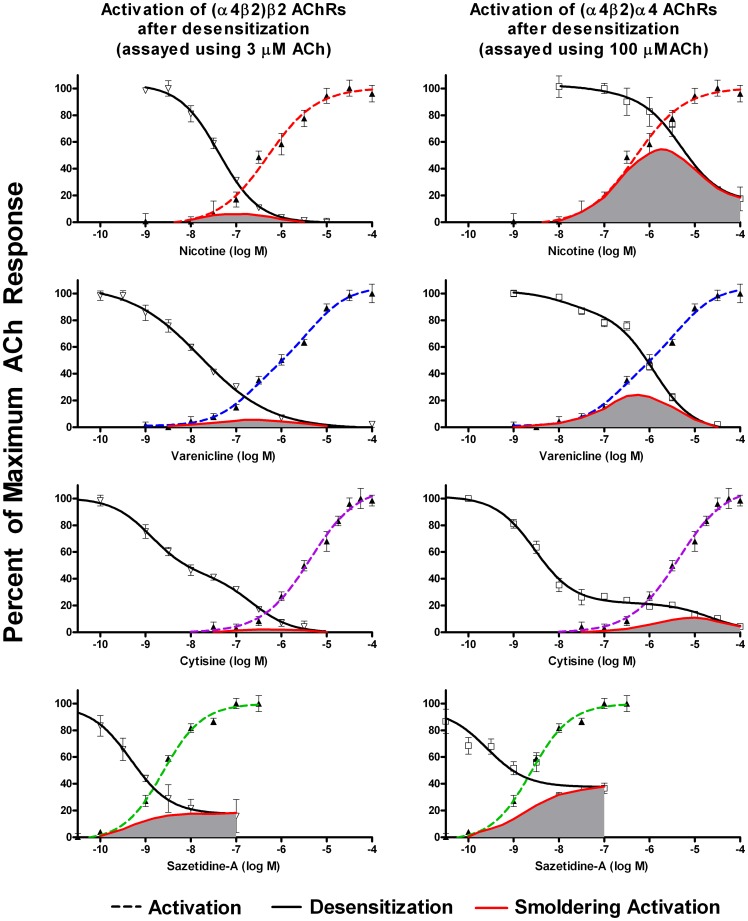
Activation and Desensitization of α4β2 AChRs by Various Agonists. Responses were measured using the FLEXStation with an indicator sensitive to changes in membrane potential. Results are expressed as a percentage of maximum fluorescence. Activity remaining after 16 hours desensitization by the indicated concentrations of agonist was assayed using 3 µM ACh (to assay function of the more sensitive stoichiometry (α4β2)_2_β2), and 100 µM ACh (to assay function of both stoichiometries). Each data point is the average of the peak fluorescence of 4–8 dose-response curves. The responses to acute application of agonists are the same as shown in Figure 2. The extent of smoldering activation (shaded area) was calculated by multiplying the extent of acute activation by the extent of sustained desensitization at each concentration. For nicotine, the area of overlap for the more sensitive (α4β2)_2_β2 stoichiometry was centered at 0.13 µM, which is a concentration typically found in smokers. Likewise for varenicline, the area of overlap for the more sensitive (α4β2)_2_β2 stoichiometry was centered at 0.16 µM, which corresponds to peak concentrations achieved in humans. Sazetidine-A was highly potent at activating as well as desensitizing α4β2 AChRs. The area of overlap for (α4β2)_2_β2 AChRs was centered at 1.5 nM. When 100 µM ACh was used for desensitization, there was a plateau on the dose response curve beginning at around 10 nM.

**Figure 4:**
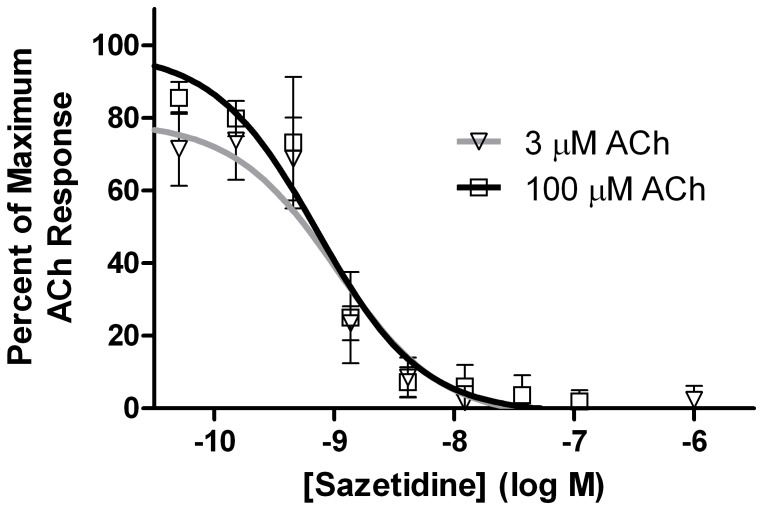
Sazetidine-A desensitization of the more sensitive (α4β2)_2_β2 stoichiometry with 3 µM and 100 µM ACh. Cells stably expressing α4β2 AChRs were further transfected with β2 subunits and cultured in nicotine as described, to enrich for the sensitive (α4β2)_2_β2 stoichiometry. Responses were measured with the FlexStation using a membrane potential sensitive indicator, and results were expressed as a percentage of maximum fluorescence. The responses to both 3 µM and 100 µM ACh overlapped, likely indicating that only the (α4β2)_2_β2 stoichiometry contributes to desensitization. The plateau on the desensitization curve with sazetidine-A on mixed stoichiometries of α4β2* (shown in Figure 3) indicates that the (α4β2)_2_α4 stoichiometry is not desensitized even at high concentrations of sazetidine-A.

**Figure 5:**
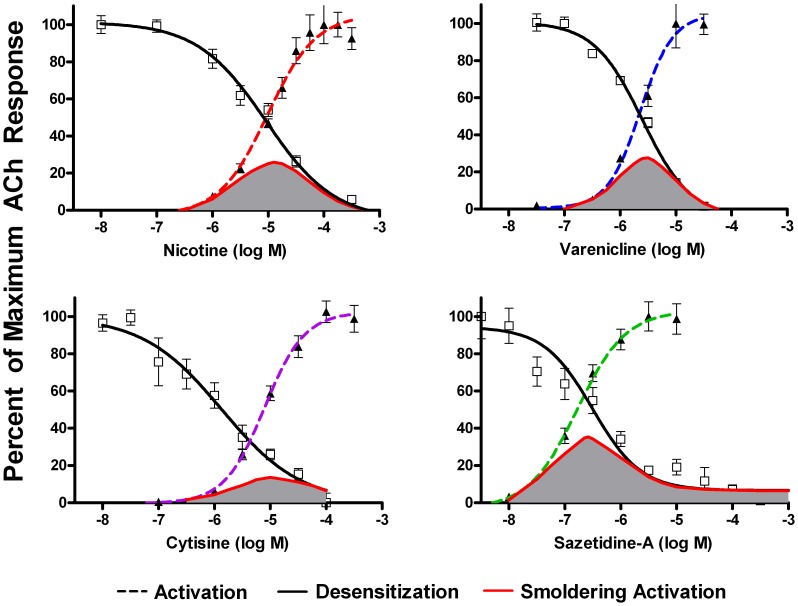
Activation and Desensitization of α3β4 AChRs by Various Agonists. Responses were measured using the FLEXStation with an indicator sensitive to changes in membrane potential. Results were expressed as a percentage of maximum fluorescence. Activity remaining after 16 hours desensitization by the indicated concentrations of agonist was assayed using 1(shaded area) was calculated by multiplying the extent of acute activation by the extent of sustained desensitization at each concentration. For nicotine and varenicline, smoldering activation of α3β4 AChRs occurs at concentrations that are above levels that can be reached in humans.

**Figure 6:**
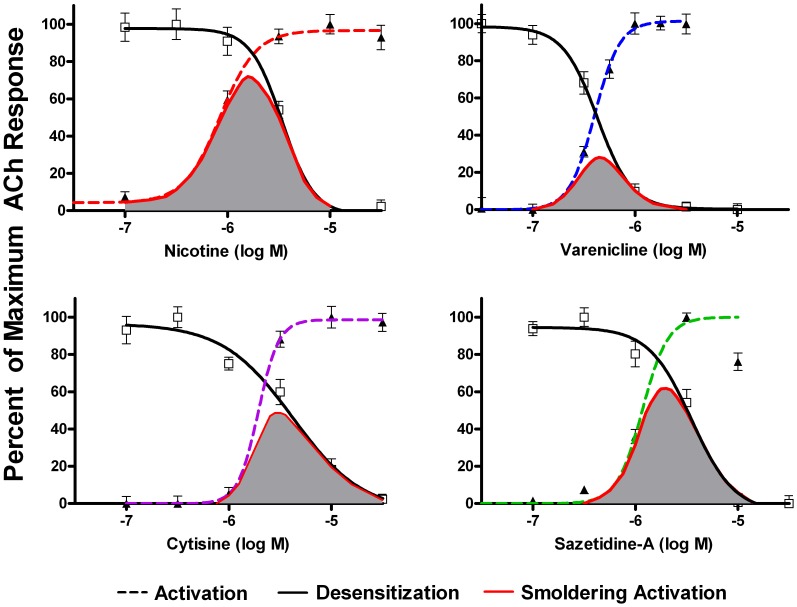
Activation and Desensitization of α7 AChRs by Various Agonists. Responses were measured using the FLEXStation with an indicator sensitive to changes in membrane potential. Results were expressed as a percentage of maximum fluorescence. Activity remaining after 16 hours desensitization by the indicated concentrations of agonist was assayed using 10 µM ACh. The extent of smoldering activation (shaded area) was calculated by multiplying the extent of acute activation by the extent of sustained desensitization at each concentration. For nicotine, the intercept of the activation and desensitization curves was 1.7 µM (well above the clinically achievable range). However, for varenicline, the intercept of the activation and desensitization curves was 0.4 µM, a concentration which can be reached with therapeutic doses of this drug.

To assess desensitization, cell lines expressing human AChRs were incubated overnight with a range of concentrations of agonists, and responses to ACh were then measured. For α4β2* AChRs, activity was assayed using 3 µM ACh (to assay function of the more sensitive (α4β2)_2_β2 stoichiometry), and 100 µM ACh (to assay function of both stoichiometries). For the other AChRs, saturating concentrations of ACh were applied (1.0 mM for α3β4 and 10 µM for α7). Responses for the three different AChRs are shown in Figures 3, 4, 5, 6, along with the dose response curves for activation (the same as shown in Figure 2). The range of concentrations at which both sustained activation and desensitization can occur (“smoldering activation”) was calculated by multiplying the acute response to agonists at each concentration by the fractional response remaining after desensitization.

The results for α4β2 are shown in Figure 3. The intercept of the activation and desensitization curves is shown in Table 1. For nicotine, the area of overlap of the activation and desensitization curves (using 3 µM ACh) was centered at 0.13 µM (summarized in Table 1). This is within the range of nicotine concentrations found in the blood of smokers (0.058–0.34 µM [43] ). The extent of calculated smoldering activation at a clinically relevant concentration of 0.1 µM nicotine was 6% of maximum response for the (α4β2)_2_β2 stoichiometry and 18% for the (α4β2)_2_α4 stoichiometry. Nicotine is capable of sustaining a calculated smoldering response of 54% of maximum response on the (α4β2)_2_α4 stoichiometry. However, this requires a nicotine concentration of 1.8 µM, which far exceeds concentrations sustained in smokers. Likewise, for varenicline, the area of overlap of the activation and desensitization curves (using 3 µM ACh) was centered at 0.16 µM. Levels of 0.4 µM are reached in patients on therapeutic doses of this drug [42]. Smoldering activation by varenicline or cytisine is likely mediated by the (α4β2)_2_α4 stoichiometry. For example, at a varenicline concentration of 1 µM, >90% of (α4β2)_2_β2 AChRs are desensitized, whereas ∼50% of (α4β2)_2_α4 AChRs are desensitized. At 1 µM varenicline, the smoldering activation is 4.3% of the maximum response for (α4β2)_2_β2 AChRs compared to 23% for (α4β2)_2_α4 AChRs. Desensitization is more likely to account for the effectiveness of these agonists in smoking cessation than is activation, since knock out of α4 or β2 (but not α7) eliminates nicotine self-administration [6].

For cytisine the area of overlap of the activation and desensitization curves for α4β2 sites (with 3 µM ACh) was centered at 0.45 µM. When desensitization by cytisine was assayed with 100 µM ACh, there was a plateau on the dose response curve for cytisine concentrations above 10 nM.

For sazetidine-A the area of overlap of the activation and desensitization curves with 3 µM ACh centered around 0.0015 µM. There was also a plateau on the desensitization curve for α4β2 AChRs with sazetidine-A using 100 µM ACh, suggesting that sazetidine-A desensitized the response of the (α4β2)_2_β2 but not the (α4β2)_2_α4 stoichiometry. To resolve the contributions of the two stoichiometries to the effects of sazetidine-A, we transfected the α4β2* expressing cell line with additional β2 subunits, and then cultured the cells in the presence of 0.5 µM nicotine in order to express predominantly the (α4β2)_2_β2 stoichiometry. As shown in Figure 4, the desensitization curves for sazetidine-A, using both 3 µM and 100 µM ACh overlapped. These curves are very similar to the one shown in Figure 3 for sazetidine-A on mixed stoichiometries of α4β2 tested with 3 µM ACh (which activates predominantly the (α4β2)_2_β2 stoichiometry). This indicates that sazetidine-A desensitizes only the (α4β2)_2_β2 stoichiometry. The plateau on the dose response curve for 100 µM ACh with mixed stoichiometries of α4β2* likely indicates a lack of agonist and desensitizing activity of sazetidine-A on the less sensitive (α4β2)_2_α4 stoichiometry. Carbone *et al.* [30] reported that sazetidine-A is a full agonist at (α4β2)_2_β2 AChRs but had <1% efficacy on the (α4β2)_2_α4 stoichiometry. Sazetidine-A may not be able to bind, activate or desensitize the third ACh binding site present at the α4/α4 interface in the (α4β2)_2_α4 stoichiometry [4], [5]. Sazetidine-A has by far the highest affinity of these agonists at the α4β2 binding sites and is exceptionally potent at inhibiting nicotine self-administration in rats [12]. This implies that inhibition of nicotine self-administration can be mediated by desensitizing α4β2* AChR responses through their α4β2 binding sites. The desensitizing effects of sazetidine-A are known to persist long after brief acute activation [44].

For all of the tested agonists, α3β4 AChRs were much less sensitive to both activation and desensitization than were α4β2 AChRs (Figure 5). The areas of overlap for the nicotine and varenicline activation and desensitization curves correspond to much higher drug levels than can be achieved in humans.

As shown in Figure 6, the activation and desensitization curves for α7 AChRs were much steeper than for either α4β2 or α3β4, as expected since α7 AChRs have five ACh binding sites acting cooperatively to activate this AChR (rather than two for (α4β2)_2_β2 and α3β4, or three for (α4β2)_2_α4). For nicotine, the area of overlap of the activation and desensitization curves for α7 corresponds to concentrations of nicotine that are higher than can be reached in humans, with an intercept of the nicotine activation and desensitization curves of 1.7 µM. However, for varenicline, the area of overlap of the activation and desensitization curves for α7 corresponds to concentrations that are within a clinically achievable range, with the intercept of the curves at 0.4 µM.

### Smoldering Activation Following Acute Desensitization

**Figure 7:**
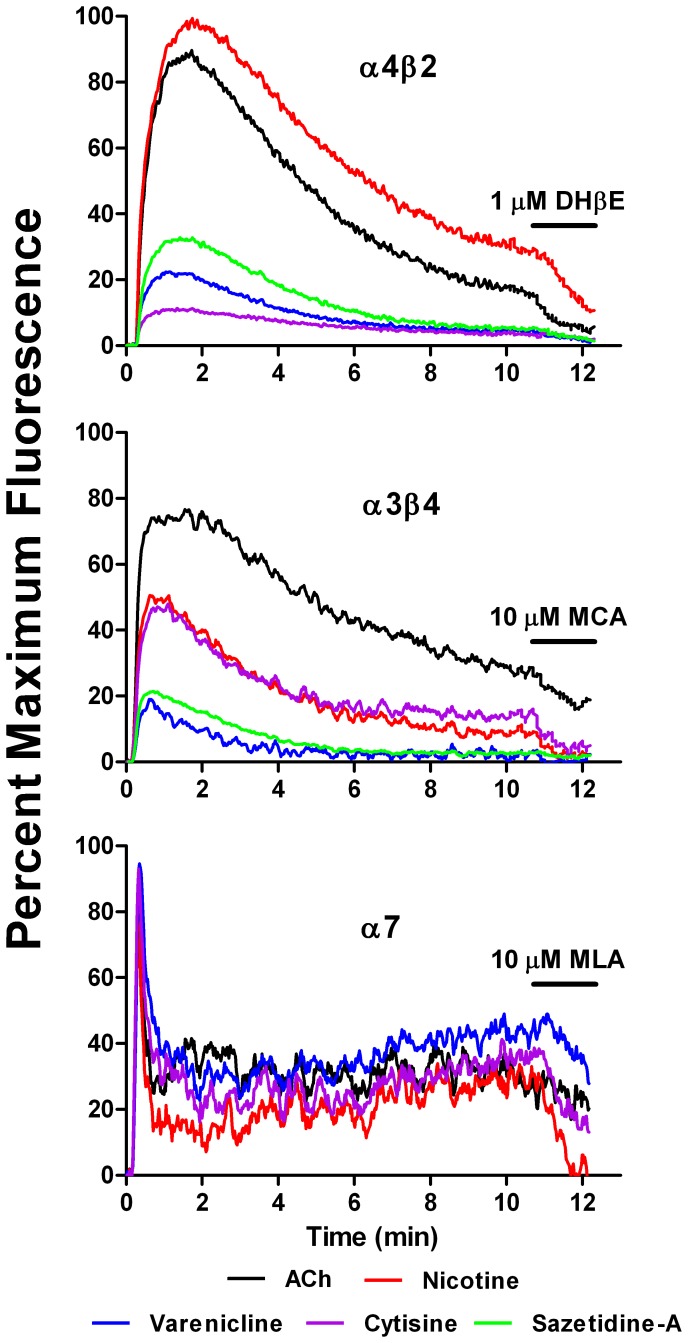
Short Term Desensitization of α4β2, α3β4 and α7 AChRs by Various Agonists. Responses of α4β2, α3β4 and α7 AChRs to the acute application of ACh (100 µM), nicotine (16 µM), varenicline (4 µM), cytisine (16 µM) and sazetidine-A (62.5 nM) were measured using the FlexStation as described. These drug concentrations were selected because they gave maximum sustained responses to these agonists. Results were expressed as a percentage of maximum response to ACh. Responses were monitored for 10 minutes, and then specific antagonists were added and responses recorded for another two minutes. The antagonists were dihydroβerythroidine (DHβE) (1 µM) for α4β2, mecamylamine (MCA (10 µM) for α3β4 and methyllycaconitine (MLA) (10 µM) for α7. These concentrations of antagonists were selected because they were sufficient to inhibit responses to the tested agonists without causing activation themselves.

We evaluated the kinetics of responses over several minutes to concentrations of the various agonists that gave maximum sustained responses. The results were expressed as a percentage of the maximum response to ACh. As shown in Figure 7, for α4β2 and α3β4 AChRs, following acute activation and partial desensitization, there was a low level of sustained activation lasting at least 10 minutes. This sustained response was abrogated by the application of specific antagonists after 10 minutes (dihydroβerythroidine (DHβE) (1 µM) for α4β2 or mecamylamine (MCA) (10 µM) for α3β4). For α7 AChRs, the initial activation and desensitization was more rapid than for α4β2 or α3β4 AChRs. The residual response after 10 minutes was abrogated by the application of the α7 antagonist methylycaconitine (MLA) (10 µM).

Thus, small but significant smoldering responses can be maintained for a period of minutes after acute activation. With α3β4 and α7 subtypes, these effects may not be significant at drug concentrations obtained *in vivo*. With α4β2 subtypes, small but significant effects may occur *in vivo,* and may contribute to nicotine addiction.

## Discussion

In this study, we utilize human AChRs cloned in human cells to examine the dual effects of activation and desensitization by nicotinic agonists. These transfected cell lines have advantages over AChRs expressed in *Xenopus* oocytes, which can retain nicotine and other agonists, and release them slowly, making it difficult to assess desensitization [45]. This is not an issue with HEK cells, which are much smaller than oocytes and lack their internal yolk compartments or other reservoirs that may account for tertiary amine uptake. Our binding studies with nicotine and epibatidine to live AChR-expressing HEK cells show that unbound agonists are easily washed away, indicating that these cells to not retain tertiary amines (unpublished results).

It has not previously been possible to measure function of α7, because levels of expression were too low for functional assays. We have overcome this limitation by co-transfection with RIC-3 and by the use of chemical chaperones [25]. Human cell lines expressing specific AChR subtypes can be used for screening and evaluating novel compounds with activity on these AChRs.

Although nicotine, varenicline, cytisine and sazetidine-A are all agonists, their behavioral effects may depend as much on desensitization as activation. Most *in vitro* studies of nicotinic agonists have examined the acute effects of these drugs over seconds to minutes. It is unclear how this relates to the *in vivo* setting, where the drugs are present for hours or days. If the major mechanism by which these partial agonists inhibit nicotine self-administration is desensitization, then this clinical effect may depend primarily on their binding affinity, rather than EC50 or efficacy.

Cytisine is a plant alkaloid used predominantly in Europe as an aid for smoking cessation [46]. It is a partial agonist on α4β2 AChRs [47]. However, its clinical utility has been limited by poor absorption and limited brain penetration. Using cytisine as a lead compound, Coe et al. synthesized a series of α4β2 partial agonists. One of these compounds, varenicline, was selected for further development because of its improved potency and efficacy [11]. Clinically, varenicline has been shown to improve long-term smoking cessation rates compared to unassisted quit attempts or bupropion (see [48] for review). It is now widely used for smoking cessation.

Sazetidine-A is a novel nicotinic receptor ligand that is highly selective for α4β2 AChRs [44]. It has potential applications for treating nicotine addiction [12], as well as depression [15], [16], [49] and pain [14], [50]. Initially it was reported to desensitize α4β2 AChRs in the absence of activation, but did not appear to either activate or desensitize rat α3β4 AChRs [44]. However, subsequently Zwart *et al.* [51], using *Xenopus* oocytes expressing human α4 and β2 subunits, found that sazetidine-A was a potent agonist for both α4β2 stoichiometries. It was a full agonist on the (α4β2)_2_β2 stoichiometry but had only 6% activity on (α4β2)_2_α4. Using pentameric concatenated (α4β2)_2_β2 and (α4β2)_2_α4 AChRs expressed in *Xenopus* oocytes, Carbone *et al.* [30] found that sazetidine-A was a full agonist on (α4β2)_2_β2 but was a partial agonist with very low efficacy on (α4β2)_2_α4 AChRs.

We found that varenicline, cytisine and sazetidine-A are partial agonists on the mixture of α4β2 AChR subtypes, but full agonists on α3β4 and α7 AChRs. While varenicline and cytisine are partial agonists on α4β2 AChRs, they are capable of fully desensitizing these AChRs to the effects of ACh. On the other hand, for sazetidine-A, full desensitization was not reached even at high concentrations, presumably because this drug has no agonist activity on the α4/α4 ACh binding site of (α4β2)_2_α4 AChRs. Varenicline, cytisine and sazetidine-A also partially upregulate α4β2 AChRs relative to nicotine (data not shown).

Acute activation of AChRs occurs within seconds of application of the agonist and is followed by acute desensitization. In the continued presence of agonist over several minutes, there is a low level of residual activation, which can be blocked by the application of specific antagonists. In the presence of agonist over many hours, there is complete desensitization of all the tested AChR subtypes, with the exception of sazetidine-A on the (α4β2)_2_α4 stoichiometry.

We propose that the area of overlap of the activation and desensitization curves defines the range of concentrations over which smoldering activation can be sustained. For α4β2 AChRs, the range of smoldering activation for nicotine and varenicline corresponds to concentrations that can be achieved clinically. However, for nicotine, the range of smoldering activation for α3β4 and α7 AChRs exceeds concentrations that can be reached in humans. For varenicline the range of smoldering activation for α3β4 AChRs exceeds clinically achievable levels. However, for α7 the range of smoldering activation corresponds to drug levels that can be reached clinically. This suggests that varenicline may have a clinical effect on α7 AChRs, which could contribute to the undesirable side effects of this drug.

The α4β2 cell line has a mixture of (α4β2)_2_β2 and (α4β2)_2_α4 stoichiometries. In order to distinguish the effects of the two stoichiometries on the desensitization of α4β2 AChRs, we used two concentrations of ACh, namely 3 µM (to detect effects on the high sensitivity stoichiometry), and 100 µM (to detect effects on both stoichiometries). Recent reports indicate that the low sensitivity (α4β2)_2_α4 stoichiometry has a third ACh binding site at the interface between adjacent α4 subunits, resulting in an intrinsic bimodal concentration response curve with an additional low sensitivity component to the response [4], [5]. Because we did not examine pure populations of (α4β2)_2_α4 AChRs, we were unable to detect this.

As expected, the agonist effect of nicotine on α4β2 AChRs was bimodal. For the more sensitive stoichiometry of α4β2, the EC50 for activation (0.18 µM) and the intercept of the activation and desensitization curves (0.13 µM) correspond to levels of nicotine that are clinically relevant (see below). On the other hand, the EC50’s and the intercepts of the activation and desensitization curves of nicotine for α3β4 and α7 AChRs are well above the range of concentrations that are achieved in smokers, indicating that these AChRs are unlikely to be involved with the reinforcing properties of nicotine. This is consistent with studies in transgenic mice showing that α7 does not contribute to nicotine self-administration, whereas the α4 and β2 subunits are both necessary and sufficient to maintain nicotine self-administration [6].

Apart from our results on (α4β2)_2_β2 for desensitization by sazetidine-A, we only examined mixed stoichiometries of α4β2. The selection of 3 and 100 µM ACh for desensitization of α4β2 AChRs may not completely separate the effects of the high and low sensitivity stoichiometries. While the use of chimeric or concatameric AChRs may separate the effects of the different stoichiometries of α4β2 AChRs, cell lines with a mixture of stoichiometries may be more representative of *in vivo* effects.

In active smokers, the majority of α4β2 AChRs in the brain are saturated [52], and thus are likely in a desensitized state. There is no information in humans on levels of nicotine in the brain during active smoking, but they are likely to be significantly higher than blood levels. Peak nicotine concentrations in venous blood of heavy smokers vary from 9.4–55.1 ng/ml (0.058–0.34 nM) [43].

At the clinically relevant nicotine concentration of 0.1 µM, the extent of smoldering activation was higher for (α4β2)_2_α4 (18%) than for (α4β2)_2_β2 (6%). This indicates that the stoichiometry which is less sensitive to acute activation is more sensitive to smoldering activation by nicotine. This may be due to the fact that the amplitude of response of the (α4β2)_2_α4 stoichiometry is 4–11 fold greater than that of (α4β2)_2_β2, probably as a result of greater probability of channel opening when three ACh binding sites are occupied [4], [5].

Our results show that cytisine is a partial agonist on α4β2 and a full agonist on α3β4 and α7, confirming what others have found [53]. The EC50 for cytisine on α4β2 was 5.5 µM. It is uncertain whether this concentration is clinically relevant, as drug levels of cytisine that can be reached in humans are not yet defined [54]. Using concatameric as well as unlinked α4β2 AChRs, Carbone *et al.* found that cytisine was a partial agonist on (α4β2)_2_α4, but was inactive on (α4β2)_2_β2 [30].

Peak varenicline levels of 0.48 µM are reached after 14 days on a standard dose of 1 mg twice daily [55]. We found that the EC50 for the more sensitive stoichiometry of α4β2 was 0.2 µM, which is within the therapeutic range, accounting for the therapeutic efficacy of this drug in treating nicotine addiction. The EC50 for α7 was 0.4 µM, also within the therapeutic range. The effect of varenicline on α7 AChRs may account for some of the toxicity of this drug. Nausea, which is a dose-limiting toxicity of varenicline, probably results from activation of 5HT_3_ receptors [56]. The cause of the rare psychotic effects of varenicline which have led to the black box warning are unclear.

We found that sazetidine-A was a partial agonist on α4β2 and a full agonist on α3β4 and α7 AChRs. It was highly selective for α4β2 AChRs, with an EC50 of 0.023 µM, compared with 0.17 µM for α3β4 and 1.2 µM for α7. However, using this fluorescence assay, we detected much greater activity of sazetidine-A on human α3β4 and α7 AChRs than did Liu *et al.* [57] with a rubidium efflux assay on rat α3β4 and α7 AChRs. Using transient transfection of β2 to the α4β2 expressing cell line we were able to resolve the effects of sazetidine-A on the two α4β2 stoichiometries. We found that sazetidine-A desensitizes only the (α4β2)_2_β2 stoichiometry. The differential effect on the two stoichiometries may explain the discrepant reports in the literature regarding whether or not sazetidine-A can activate α4β2 AChRs.

The results reported here allow us to speculate on the effects of prolonged presence of these agonists on endogenous cholinergic signaling *in vivo* as well as modulation of the effects of nicotine in smokers. Since varenicline is a partial agonist with greater affinity than nicotine and consequently more potency at desensitizing, the smoldering activation produced by nicotine on α4β2 AChRs will be reduced in the presence of varenicline. The net effect of varenicline will be antagonistic to both the effects of nicotine and endogenous ACh signaling.

Cytisine has lower efficacy than varenicline on α4β2 but also has lower affinity and consequently less potency at desensitizing. The net effect of cytisine will be antagonistic to both the effects of nicotine and endogenous ACh and it will decrease smoldering activation by nicotine.

Sazetidine is a partial agonist with much higher affinity than either varenicline or cytisine for (α4β2)_2_β2 and it does not desensitize (α4β2)_2_α4. Therefore, it has a very potent net desensitizing effect on the (α4β2)_2_β2 stoichiometry while allowing nicotine to cause smoldering activation or desensitization of the (α4β2)_2_α4 stoichiometry.

On α3β4 AChRs, nicotine is expected to produce little activation or desensitization at concentrations typically sustained in smokers. Varenicline at submicromolar concentrations will also have limited effect. Cytisine should cause significant desensitization, but little agonist activity, at submicromolar concentrations. Sazetidine at submicromolar concentrations will cause significant smoldering activation as well as desensitization, thereby differing significantly from varenicline and cytisine.

On α7 AChRs, nicotine at concentrations sustained in smokers should have little agonist or desensitizing effect. Varenicline should cause significant smoldering activation and desensitizing effects at the concentrations used for smoking cessation therapy. This might contribute to the off target effects which have given it a black box warning of psychopathological effects in some smokers. Cytisine should have little effect on α7 at therapeutic doses. However, sazetidine could have very substantial smoldering agonist effects on α7 at concentrations that would be therapeutically significant. This could produce significant off target effects.

In summary, we have defined the range of concentrations of nicotinic agonists and partial agonists which can sustain smoldering activation of human α4β2, α3β4 and α7 AChRs. Further studies are needed to determine the role of smoldering activation not only in nicotine addiction but also in the therapeutic effects of nicotinic agonists and partial agonists.
